# Effects of hydrolyzable tannin extract obtained from sweet chestnut wood (*Castanea sativa* Mill.) against bacteria causing subclinical mastitis in Thai Friesian dairy cows

**DOI:** 10.14202/vetworld.2021.2427-2433

**Published:** 2021-09-20

**Authors:** Tipwadee Prapaiwong, Wuttikorn Srakaew, Chalong Wachirapakorn, Chaiwat Jarassaeng

**Affiliations:** 1 Department of Animal Science, Faculty of Agriculture, Khon Kaen University, Khon Kaen 40002, Thailand; 2 Division of Theriogenology, Faculty of Veterinary Medicine, Khon Kaen University, Khon Kaen 40002, Thailand.

**Keywords:** hydrolyzable tannins, pathogen, subclinical mastitis, Thai Friesian dairy cows

## Abstract

**Background and Aim:**

Hydrolyzable tannins are an important group of secondary plant metabolites, which are known for antimicrobial activity. This study aimed to assess the efficiency with which a hydrolyzable tannin extract from sweet chestnut wood (*Castanea sativa* Mill.) could inhibit mastitis-causing bacteria *in vitro*.

**Materials and Methods:**

The negative control used was sterile water, and the positive controls were penicillin and gentamicin. The treatments included five concentrations of hydrolyzable tannins (63, 190, 313, 630, and 940 mg/mL). In cows with subclinical mastitis, the bacteria causing the disease were isolated and identified. Then, the antibacterial activity of the hydrolyzable tannin extract was assessed by the disk diffusion method, by determining the minimum inhibitory concentration (MIC) and by determining the minimum bactericidal concentration (MBC).

**Results:**

Penicillin inhibited (p<0.01) the growth of *Staphylococcus aureus*, *Streptococcus uberis*, and *Pseudomonas aeruginosa* but could not inhibit (p>0.05) the growth of *Streptococcus agalactiae*, *Escherichia coli*, and *Klebsiella pneumoniae*. However, gentamicin and hydrolyzable tannins could inhibit (p<0.01) all isolated bacteria. Increasing the concentration of hydrolyzable tannin extract resulted in a quadratic increase in the inhibition zone diameter of *S. aureus* and *S. agalactiae* and a linear increase in the inhibition zone diameter of *E. coli*, *K. pneumoniae*, and *P. aeruginosa*. In addition, 630 and 940 mg/mL of hydrolyzable tannin extract showed the highest antibacterial activity against *S. agalactiae* and *E. coli* (p<0.01), while 940 mg/mL concentration had the highest antibacterial activity against *K. pneumoniae* (p<0.01). The MIC and MBC of the extract were 27.3-190 mg/mL and 58.8-235 mg/mL, respectively, with the MBC: MIC ratio being 2:1.

**Conclusion:**

The antimicrobial activity of the hydrolyzable tannin extract against subclinical mastitis bacteria was comparable to the antibiotics (positive controls) at concentrations over 630 mg/mL. Although these *in vitro* findings are promising, further research is needed to determine whether hydrolyzable tannins could be used to control or prevent subclinical mastitis in dairy cows.

## Introduction

Globally, subclinical mastitis is a persistent problem and the most economically disruptive type of mastitis in dairy farms. It is difficult to detect due to non-visible changes to the udder or in the milk [1-3]. Subclinical mastitis may result in an increased number of inflammatory cells in the milk [4] as well as causes a decrease in milk supply (10-20%) and quality and an increase in somatic cell counts [5,6]. This disease is challenging to diagnose, highly prevalent, associated with long-term devastating effects, and causes the most financial losses to herders [7,8]. Thus, subclinical mastitis is widely known as an extremely difficult to control infection. In addition, outbreaks of the disease are increasing, resulting in increased likelihood of rate of culling and a higher cost of veterinary treatment [9]. For example, Turk *et al*. [10] showed that 23% of cows leaving the herds too early are culled because of udder health problems. Similarly, Yadav [11] reported that 60-70% of economic losses incurred by dairy farmers were associated with subclinical mastitis.

Due to poor management techniques and a lack of knowledge and understanding of subclinical mastitis among farmers, the prevalence of this disease was reported to be as high as 62.8% in certain provinces of Northeast Thailand [12]. The predominant microorganisms isolated from infected smallholder dairy cows in the Khon Kaen Province (sampling area of the present study) were *Streptococcus* spp. and coagulase-negative staphylococci [13]. At present, of all the antimicrobials administered to dairy animals, approximately 60-70% are used for preventing and treating bovine mastitis [14]. As a consequence, Cobirka *et al*. [15] observed a concerning problem that pathogens isolated from milk were able to resist antimicrobial agents such as streptomycin, neomycin, cephalexin, and penicillin [15]. High resistance was observed in *Streptococcus uberis* and *Escherichia coli* isolates (86% and 79%, respectively) [15]. Holko *et al*. [16] reported that approximately 62% of isolated mastitis-causing bacteria were resistant to at least one antimicrobial agent. In Thailand, cloxacillin and gentamicin were being widely used for treating bovine mastitis, and isolates of *Staphylococcus aureus* resistant to both these agents were detected [17,18]. In this context, an alternative treatment for subclinical mastitis is required. However, there are limited data regarding such therapies in Thailand. Within the next few years, hydrolyzable tannins are likely to become an important component of antimicrobial agents due to their natural origin as well as safety environment.

Hydrolyzable tannins are obtained from plant derivatives that include both ellagitannins and gallotannins [19]. These have been used as feed additives [20-22] due to their biological properties as antibacterial agents. However, hydrolyzable tannins can also inhibit extracellular microbial enzymes and led to deprivation of their substrates, as well as decrease the intake of essential microbial minerals such as iron and zinc [23-27].

The aim of this study was to investigate and assess the efficacy of a hydrolyzable tannin extract of sweet chestnut wood (*Castanea sativa* Mill.) in inhibiting pathogens isolated from the milk of Thai Friesian dairy cows with subclinical mastitis.

## Materials and Methods

### Ethical approval

The Animal Ethics Committee of Khon Kaen University, based on the Ethics of Animal Experimentation of the National Research Council of Thailand, approved the experimental procedure (Record no IACUC-KKU-86/2560).

### Study period, farm and milk sampling

In this study, milk was collected from smallholder dairy farms in Khon Kaen Province, Thailand (16°34′ N, 102°46′ E) from January to June 2016. The farmers milked cows manually using a bucket-type milking machine twice a day: In the morning (6.00-8.00 am) and afternoon (2.30-5.00 pm). Milk samples were collected from 24 Thai Friesian dairy cows aged 3-6 years. Teats were cleaned aseptically before milk sample collection.

Samples were collected from cows that were not on any antibiotic treatment for at least 3 weeks. Screening for subclinical mastitis was performed during morning milking using the California mastitis test (CMT) [28]. A CMT score of more than 1 indicated subclinical mastitis. Subsequently, 15 mL of milk sample was collected aseptically from each cow in accordance with the National Mastitis Council guidelines [29]. The milk was immediately cooled on ice to 4°C and transported for bacterial culture and analysis to the microbiology laboratory at the Animal Hospital, Faculty of Veterinary Medicine, Khon Kaen University.

### Bacterial identification

A microbiological examination was performed within 2 h after milk collection. Briefly, 10 mL of milk was spread onto two selective media: 5% bovine blood agar plates (for culturing Gram-positive ­bacteria) and MacConkey’s agar plates (for culturing Gram-negative bacteria). Subsequently, the plates were incubated at 37°C for 24-48 h, and bacterial colonies were then isolated. Each isolate was identified using colony morphology and Gram staining. The Gram-positive bacteria *S. aureus*, *S. uberis*, and *Streptococcus agalactiae* and the Gram-negative bacteria *E. coli*, *Klebsiella pneumoniae*, and *Pseudomonas aeruginosa* were identified according to the conventional biochemical tests [30,31]. *S. aureus* culture is characterized by jet black colonies surrounded by a white halo. We performed the coagulase test to confirm the presence of *S. aureus*. The long-chain, hemolysis, and esculin hydrolysis colony (dark background) specifically suggested cases of *S. uberis* isolation. Afterward, the colony was grown on an Edward media plate (media for *S. uberis*), and the colonies were picked using a wire loop then restreaked on MacConkey’s agar. The absences of growth on MacConkey’s agar suggested the presence of *S. uberis*. The appearance of a clear (transparent) zone and flat colonies with a narrow zone of b-hemolysis on blood agar indicated *S. agalactiae*, which were confirmed using the Christie–Atkins–Munch-Peterson biochemical test. All *E. coli* and *K. pneumoniae* colonies were bright pink to pink in color with mucoid production on MacConkey’s agar. Methyl red/Voges–Proskauer test was used to distinguish between *E. coli* and *K. pneumoniae*. Finally, *P. aeruginosa* was identified with the blue-green appearance of colonies. The presence of *P. aeruginosa* was then confirmed by biochemical tests, including sugar fermentation test and oxidase reaction.

### Antibacterial activity of hydrolyzable tannins

Polyphenols extracted from sweet chestnut wood (*C. sativa* Mill.) as a brown powder. The polyphenol crude extract contained approximately 63% hydrolyzable tannin. This powder was dissolved in 1 mL of distilled water and stored for 1 h at 25°C for use in antibacterial activity assay. The control group was treated with sterile water, which was used as the experimental negative control, whereas penicillin and gentamicin were used as positive controls. These were compared with five concentrations of hydrolyzable tannins were 63, 190, 313, 630, and 940 mg/mL. The antibacterial activity of hydrolyzable tannins was assessed by the disk diffusion method, according to the recommendations of the National Committee for Clinical Laboratory Standards (NCCLS) [32] using bacteria isolated from the milk of cows with subclinical mastitis. The obtained bacterial isolates were added at a concentration of 1×10^8^ colony-forming unit (CFU)/mL into solution tubes containing hydrolyzable tannins of different concentrations. The suspension was then immediately spread onto plates with blood Mueller-Hinton agar and Mueller-Hinton agar. For disk diffusion assay, a sterile 6 mm paper disk (Whatman, Maidstone, UK) was impregnated with 10 mg of hydrolyzable tannins. All antimicrobial disks used as positive controls (impregnated with 10 mg penicillin and 10 mg gentamicin) were obtained from Oxoid™ Ltd (Thermo Fisher Scientific, USA). The disks used as negative controls were impregnated with distilled water. Subsequently, all disks were placed on separate agar plates containing bacteria. The plates were then incubated at 37°C for 24 h.

### Minimum inhibitory concentration (MIC) of hydrolyzable tannins

The MIC assay of hydrolyzable tannins was performed using the microdilution technique in accordance with NCCLS [32]. Briefly, each concentration of the hydrolyzable tannin extract was dissolved in 1 mL Mueller-Hinton broth and diluted to achieve different concentrations of 0.06-63, 0.19-190, 0.31-313, 0.62-630, and 0.92-940 mg/mL using 2-fold serial dilutions. These solutions were then transferred into 11 sterile, capped tubes. The control group comprised a tube containing bacteria only and a tube for each concentration containing hydrolyzable tannin extract without bacteria. Penicillin and gentamicin were diluted from 0.0001 to 1 µg/mL, respectively. The bacteria were diluted by adding saline into each tube of hydrolyzable tannins to obtain a count of to 1×10^8^ CFU/mL. Then, 1 mL of sample from each tube was spread onto nutrient agar plates, which were then inoculated at 36°C for 24 h to confirm the effectiveness of hydrolyzable tannins. The lowest concentration at which bacteria were completely inhibited was established as the MIC.

### Minimum bactericidal concentration (MBC) assay of hydrolyzable tannins

The MBC is the lowest concentration at which an antibacterial agent can kill 99.9% of the initial bacterial inoculum. The MBC of hydrolyzable tannins was determined using the method reported by Spencer and Spencer [33]. Briefly, 1 mL of sample from each tube used for MIC determination was spread on nutrient agar plates, and the inoculated plates were then incubated at 37°C for 24 h. The MBC was recorded as the lowest broth dilution of hydrolyzable tannins that prevented bacterial growth on the agar plate.

### Statistical analysis

Data corresponding to each isolated bacteria were subjected to statistical analysis according to a completely randomized design using the general linear model using the SAS version 9.4 (SAS Institute Inc.^®^ Cary, NC, USA) [34]. The differences in means obtained from the respective treatments were assessed using Scheffe’s test. In addition, orthogonal polynomial contrasts were used to examine the responses to increased levels of hydrolyzable tannins. Statistical significance was assumed at p<0.05.

## Results

### Antibacterial activities

The effect of antibiotics and hydrolyzable tannin extract against the bacteria causing subclinical mastitis is shown in Table-1. *In vitro* antimicrobial activity testing, using the filter paper disk agar diffusion technique, found that the hydrolyzable tannin extract differed significantly from the antibiotics (p<0.01) with regard to inhibiting all mastitis-causing bacteria. Penicillin had the highest antibacterial activity against *S. aureus*, *S. uberis*, and *P. aeruginosa* (p<0.01), but it could not inhibit *S. agalactiae*, *E. coli*, and *K.­­ ­pneumoniae*. Gentamicin inhibited all isolated bacteria (p>0.01), albeit to a lesser extent than penicillin for *S. aureus*, *S. uberis*, and *P. aeruginosa*. In addition, concentrations of hydrolyzable tannin extract >63 mg/mL showed the ability to inhibit (p<0.01) all mastitis-causing bacteria. The diameters of inhibition zones against all isolated bacteria increased linearly (p<0.05) with increasing concentration of the hydrolyzable tannin extract. Notably, 630 mg/mL of hydrolyzable tannin extract had an antibacterial activity similar to that of gentamicin against *S. aureus*, *S. uberis*, and *P. aeruginosa*. Similarly, 190 mg/mL of the extract had an antibacterial activity similar to that of gentamicin against *S. agalactiae*, *E. coli*, and *P. aeruginosa*. Increasing the concentration of hydrolyzable tannins quadratically increased (p<0.01) the inhibition zone diameters of *S. aureus* and *S. agalactiae*, while it linearly increased (p<0.01) the inhibition zone diameters of *E. coli*, *K. pneumoniae*, and *P. aeruginosa*.

**Table 1:** Growth inhibition zones (mm) for different bacteria at a concentration of penicillin, gentamicin, and hydrolyzable tannins.

Items	Mean diameter (mm) of inhibition zone for the growth of various bacteria					

	*Staphylococcus aureus*	*Streptococcus uberis*	*Streptococcus agalactiae*	*Escherichia coli*	*Klebsiella pneumoniae*	*Pseudomonas aeruginosa*
Control	0.00^e^	0.00^e^	0.00^e^	0.00^c^	0.00^d^	0.00^e^
Penicillin	34.00^a^	33.33^a^	0.00^e^	0.00^c^	0.00^d^	19.00^a^
Gentamicin	14.66^b^	14.33^b,c^	7.33^c,d^	2.66^c^	6.00^b,c^	11.66^c^
Hydrolyzable tannins (mg/mL)						
63	5.66^d^	7.33^d^	5.00^d^	0.00^c^	0.00^d^	6.33^d^
190	7.66^d^	8.00^d^	8.66^c,d^	3.16^b,c^	4.66^c^	8.66^c,d^
313	11.16^c^	11.00^c,d^	10.00^b,c^	6.00^b^	7.33^b,c^	11.33^c^
630	14.16^c,b^	16.66^b^	16.33^a^	10.00^a^	9.66^b^	12.33^b,c^
940	13.66^c,b^	17.33^b^	13.66^a,b^	11.33^a^	15.66^a^	16.66^a,b^
SEM	0.52	0.88	0.62	0.53	0.74	0.82
p-value	<0.01	<0.01	<0.01	<0.01	<0.01	<0.01
Contrast (p-value)						
Control versus others	<0.01	<0.01	<0.01	<0.01	<0.01	<0.01
Penicillin versus gentamicin	<0.01	<0.01	<0.01	<0.01	<0.01	<0.01
Antibiotic versus hydrolyzable tannins	<0.01	<0.01	<0.01	<0.01	<0.01	<0.01
Hydrolyzable tannins						
Linear	<0.01	0.50	<0.01	<0.01	<0.01	<0.01
Quadratic	<0.05	0.75	<0.05	0.33	0.52	0.33
Cubic	<0.05	0.61	<0.05	0.28	0.08	0.15
Quartic	0.85	0.94	<0.01	0.35	0.77	0.20

0.0=No bacterial growth inhibition zone. ^a-e^Means in the same row with different superscript differ (p<0.05). SEM=Standard error of the mean

### MIC and MBC

The MIC, MBC, and MBC: MIC ratios of hydrolyzable tannins against bacteria isolated from cows with subclinical mastitis are presented in Table-2. The MIC of hydrolyzable tannins ranged from 27.3-190 mg/mL across all isolated mastitis-causing bacteria, while the MIC of penicillin and gentamicin were in the range of 0.254-0.0001µg/mL. The MBC of hydrolyzable tannins was found to be 58.8-235 mg/mL when hydrolyzable tannins were added at concentrations ranging from 63 to 940 mg/mL. The MBC of penicillin and gentamicin was between 0.625 and 0.0001 µg/mL, indicating stronger antibacterial activity than hydrolyzable tannins. The MBC: MIC ratios of hydrolyzable tannins at the concentrations of 63 and 313 mg/mL were 1:2 and at 630 and 940 mg/mL were 2:1.

**Table 2:** MIC, MBC, and MBC:MIC ratio of hydrolyzable tannins against bacteria isolates.

Items	Control	Penicillin	Gentamicin	Hydrolyzable tannins (mg/mL)				

				63	190	313	630	940
MIC								
*S. aureus*	0.0	0.0001	0.006	63.0	71.3	156.5	91.4	58.8
*S. uberis*	0.0	0.0001	0.254	63.0	95.0	78.3	119.7	117.5
*S. agalactiae*	0.0	0.0001	0.0001	31.5	35.6	39.1	151.2	117.5
*E. coli*	0.0	0.005	0.002	63.0	47.5	78.6	119.7	29.3
*K. pneumoniae*	0.0	0.0001	0.0015	63.0	190.0	156.5	151.2	117.5
*P. aeruginosa*	0.0	0.0001	0.0001	63.0	27.3	97.8	88.2	29.3
MBC								
*S. aureus*	0.0	0.0001	0.012	NA	95.0	234.8	148.1	117.5
*S. uberis*	0.0	0.0001	0.625	NA	95.0	78.3	179.6	235.0
*S. agalactiae*	0.0	0.0001	0.0001	63.0	71.3	78.3	226.8	235.0
*E. coli*	0.0	0.133	0.282	NA	NA	97.8	179.6	58.8
*K. pneumoniae*	0.0	0.0001	0.01	NA	190.0	156.5	226.8	235.0
*P. aeruginosa*	0.0	0.0001	0.0001	NA	NA	97.8	148.1	58.8
MBC:MIC ratio of hydrolyzable tannins								
*S. aureus*	-	-	-	0:1	1:1	2:1	2:1	2:1
*S. uberis*	-	-	-	0:1	1:1	1:1	2:1	2:1
*S. agalactiae*	-	-	-	2:1	2:1	2:1	2:1	2:1
*E. coli*	-	-	-	0:1	0:1	1:1	2:1	2:1
*K. pneumoniae*	-	-	-	0:1	1:1	1:1	2:1	2:1
*P. aeruginosa*	-	-	-	0:1	0:1	1:1	2:1	2:1

NA=No activity, MIC=Minimum inhibitory concentration, MBC=Minimum bactericidal concentration, *S. aureus*=*Staphylococcus aureus*, *S. uberis=Streptococcus uberis, S. agalactiae=Streptococcus agalactiae, E. coli=Escherichia coli, K. pneumoniae=Klebsiella pneumoniae, P. aeruginosa=Pseudomonas aeruginosa*

## Discussion

Penicillin is an antimicrobial agent used to treat a wide range of bacterial infections and diseases [35], particularly most streptococci (≥92%) [36]. However, in the present study, penicillin could inhibit only three of six isolated subclinical mastitis-causing bacteria, whereas gentamicin and hydrolyzable tannins could inhibit all isolated bacteria. Salih and Gibreel [37] found that gentamicin was the best antibiotic for ­treating bovine mastitis-causing bacteria. In Thailand, isolates of *S. aureus* were found to be resistant to antibiotics such as cloxacillin and gentamicin, which are commonly used for treating bovine mastitis [17,18]. Based on the findings of our study, hydrolyzable tannins could be used instead of gentamicin to treat dairy cows with subclinical mastitis.

The inhibition zone of hydrolyzable tannins was 3.16-17.33 mm in diameter, and this increased as the concentration of hydrolyzable tannins increased. The largest inhibition zone inhibition was observed at 630 and 940 mg/mL of hydrolyzable tannins against isolated mastitis-causing bacteria. The results suggest that hydrolyzable tannins at 630 and 940 mg/mL have a moderate effect on the diameter of bacterial inhibition zone. Zarin *et al*. [38] have suggested that antibacterial agents normally have an inhibition zone diameter of 11-20 mm. Min *et al*. [39] demonstrated that 2 mg/mL of hydrolyzable tannins inhibited the growth of *E*. *coli* and *S*. *aureus* and had a mean inhibition zone diameter of 15-25 mm, which was larger than that noted in the present study.

While selecting an antimicrobial drug, the MIC must be taken into consideration, as a lower MIC indicates the requirement of a lower drug concentration to inhibit microbial growth [40]. Doss *et al*. [41] suggested that the higher the MIC lowers the antibacterial activity, whereas a lower MIC resulted in higher antibacterial activity. In the present study, a high concentration of hydrolyzable tannins demonstrated a strong antibacterial activity. These findings corroborated those of Banso and Adeyemo [42], who observed a low MIC with the addition of tannin. However, the inhibition zone varied according to different factors, including plant extract sources, chemical structure, concentration, levels and species of bacteria, methodology, and interpretative criteria [43,44]. Similarly, Zarin *et al*. [38] suggested that different MIC or MBC against different bacteria were related to different bacterial groups and degrees of sensitivity to antimicrobial compounds. Schwarz *et al*. [45] recommended that comparisons of study results should only be made if these studies used similar criteria and methodology for interpretation.

In our study, MIC and MBC of the hydrolyzable tannins were lower than those of penicillin and gentamicin. Penicillin is the most common bactericidal agent for treating bovine mastitis and other diseases in dairy cattle. However*, S*. *agalactiae*, *E*. *coli*, and *K*. *pneumoniae* are highly resistant to penicillin [46,47]. In addition, *E*. *coli* and *K*. *pneumoniae* are Gram-negative bacteria that are more resistant to antibiotics than Gram-positive bacteria, while Gram-negative bacteria are more resistant to antimicrobials derived from natural extracts [48]. Therefore, large molecule drugs have no effect on Gram-negative bacteria [49].

The MBC:MIC ratios of hydrolyzable tannins at 630 and 940 mg/mL were both 2:1, indicating good antibacterial activity. According to MBC: MIC data, natural products seem to have positive antimicrobial effects compared with synthetic antimicrobial drugs [50]. Reportedly, the MBC: MIC ratio of antibacterial agents ranged from 1:1 to 2:1 and occasionally more than 2:1, indicating high antimicrobial activity similar to the bacteriostatic agents in drugs [51]. Our results were similar to those of Chung *et al*. [52], who reported that tannins function as bacteriostatic or bactericidal agents against *S. aureus*. Furthermore, hydrolyzable tannins act as antimicrobial agents that have the potential to damage the lipid bilayer membrane and microbial cell membrane, break the cell wall, and inhibit bacterial growth and extracellular microbial enzymes [53-55]. According to the findings of *in vitro* antimicrobial activity tests in this study, hydrolyzable tannins at concentrations ≥630 mg/mL are suitable to induce high antibacterial activity against subclinical mastitis-causing bacteria.

## Conclusion

Hydrolyzable tannins at 630 and 940 mg/mL showed good bacterial growth inhibition zone (13.19 and 14.72 mm, respectively). In addition, hydrolyzable tannins had a moderate zone inhibition diameter against subclinical mastitis-causing bacteria. Hydrolyzable tannins are potent antibacterial agents that can be used to control or prevent subclinical mastitis in dairy cows. However, further investigation is needed to disclose the precise mode of action of hydrolyzable tannins against mastitis.

## Authors’ Contributions

CJ, CW, and TP: Conceptualization. CJ, and TP: Methodology. TP and WS: Formal analysis. TP and CJ: Writing and original draft preparation. TP and CJ: Writing, review, and editing. CW and CJ: Supervision. CJ: Project administration. All authors read and approved the final manuscript.
